# Ethnic Profile of Patients Discharged From a First Episode Psychosis Service in Derby City and South County

**DOI:** 10.1192/bjo.2025.10510

**Published:** 2025-06-20

**Authors:** Mahendra Kumar, Remon Mosaad

**Affiliations:** Derbyshire Healthcare Foundation Trust, Derby, United Kingdom

## Abstract

**Aims:** The Early Intervention for Psychosis (EIP) service in Derby City and Derbyshire South County provides care for individuals aged 14–65 experiencing a first episode of psychosis. Derby City (Census 2021 population: 261,400) is ethnically diverse, with White residents forming 73.8%, Asian residents 18.1% (including mixed White/Asian, and Arab), and Black residents 6.1% (including White and Black Caribbean, mixed White/Black, and African). In contrast, Derbyshire South County (Census 2021 population: 349,000) has a predominantly White population (95.2%), with Asian residents at 2.8%, Black residents at 1.4%, and other ethnic groups at 0.5%.

Aim was to ascertain the ethnic profile of patients discharged from the EIP service in Derby City and Derbyshire South County, comparing these findings with respective census data.

**Methods:** All patients discharged from the EIP service between 1 April 2023 and 1 April 2024, who were under the service for more than 3 months and typically not more than 3 years, were included. Data on ethnicity was retrospectively collected from clinical records, recorded in an Excel spreadsheet, and analysed to identify disparities compared with census demographics.

**Results:** In Derby City, White patients were under-represented at 60.87% compared with 73.8% in the census. Asian patients were over-represented at 21.74% versus 18.1%, and Black patients accounted for 17.39% of discharges compared with 6.1%. Other ethnic groups were absent (0%) compared with 2.0% in the census.

In Derbyshire South County, White patients represented 61.36% of discharges, lower than 95.2% in the census. Asian patients were over-represented at 27.27% compared with 2.8%, and Black patients at 11.36% versus 1.4%. No representation was observed from other ethnic groups, despite a 0.5% census presence.

**Conclusion:** The study highlights disparities in the ethnic profile of discharged EIP patients. Asian and Black populations were consistently over-represented, while White populations were under-represented, especially in Derbyshire South County. The absence of other ethnic groups raises concerns about service access. Further investigation is needed to explore factors such as socio-economic influences, cultural perceptions of mental health, referral pathways, and potential systemic biases.

